# Regulating the relationship between physicians and pharmaceutical companies: a qualitative and descriptive analysis of the impact of Israeli legislation

**DOI:** 10.1186/s13584-017-0170-3

**Published:** 2017-09-26

**Authors:** Rachel Nissanholtz-Gannot, Ariel Yankellevich

**Affiliations:** 1 Department of Health System Management, Ariel University, University Hill, 40700 Ariel, Israel; 2 Myers-JDC-Brookdale Institute, Jerusalem, Israel; 3 Department of Sociology and Anthropology, Ben Gurion University of the Negev, Be’er Sheva, Israel

**Keywords:** Physician-pharmaceutical companies’ relationship, Payments, Stakeholders, Transparency, Legislation

## Abstract

**Background:**

The Israeli National Health Insurance Law was amended in 2010 to require the disclosure of payments above 2500 NIS from pharmaceutical companies (PCs) to medical personnel and organizations. We examined if the law had an impact on the relationship between physicians in the Israeli health system and the pharmaceutical industry.

**Methods:**

We conducted 42 in-depth semi-structured interviews with representatives of relevant stakeholders regarding the effects and extent of the law and the interviewees’ attitudes about regulating the relationship between physicians and PCs. In addition, we analyzed reports on payments from PCs to various components of the health system.

**Results:**

The majority of interviewees agreed that transparency is important to the relationship between PCs and physicians and none of them opposed the disclosure of payments. Most interviewees reported to have witnessed a change in the regulatory climate of the relationship between PCs and physicians, prompted mostly by self-regulatory measures of the pharmaceutical industry. The most significant change in this relationship appeared to be the enactment of contractual relations between PCs and physicians. There was a pervasive feeling that self-regulation is more effective than state regulation. The impact of the law on the behavior of individual physicians was claimed to be limited at best. Suggested causes were lack of awareness of the law, particularly among physicians; ambiguous definition of “payments” and loopholes in the law that attract other forms of remuneration to physicians and lack of enforcement of the law. According to reports published by the Ministry of Health, Pharma Israel, and the Israeli Medical association, although there had been some disclosure of payments by both donors and beneficiaries, there were inconsistencies between the total payments disclosed by PCs and those disclosed by their beneficiaries.

**Conclusions:**

There is a broad agreement that transparency is important to the PCs-physicians relationship. In addition, it seems that there was a change in the regulatory climate of that relationship; however, the feeling among the stakeholders is that self-regulation is more effective than state regulation. Therefore, efforts should focus on enforcement of the law and deterrence of its violations, possibly by investigating discrepancies between disclosed payment by donors and beneficiaries. The law should be amended to close loopholes. Furthermore, there should be periodic follow up of relevant databases, and relevant stakeholders should be interviewed in order to seek feedback and identify problems in implementation.

## Background

Conflicts of interest often arise in the practice of medicine. A specific example is the relationship between physicians and pharmaceutical companies (PCs). This relationship is not governed by a clear code of ethics [1] and there has been much debate on how to deal with it and regulate it [2–5]. Several multinational PCs and their umbrella organizations as well as medical associations have adopted ethical codes that define and regulate their relationships with physicians [6–12]. In addition, at the state regulation level, many countries have non-specific laws that also apply to the relationship between healthcare personnel including physicians and PCs, such as anti-bribery laws [8]. More recently, several countries, such as the USA, France, Portugal Slovakia, Belgium, Denmark, Italy, Germany, Spain and Israel, have passed transparency (“sunshine”) acts and rules that are specific to this relationship [13]. For example, in the USA, the Physician Payment Sunshine Act, a part of the 2010 Affordable Care Act, mandates that all manufacturers of drugs, medical devices and medical supplies, who have at least one product covered by Medicare or Medicaid, disclose payments and gifts they have made to physicians and teaching hospitals. The Centers for Medicare and Medicaid Services receive reports from industry on relevant financial interactions and make the information available on the “Open Payments” website. In France, the Bertrand Act requires companies to make public any “benefits” given to health professionals (e.g., meals, lodging, equipment payments) and “conventions,” or agreements, between companies and providers (e.g., speaking at conferences, research payments involvement in clinical trial work, training) [13]. The aim of publically releasing PCs’ payments is to promote overall transparency and to provide patients with the ability to assess where their physician’s bias and financial interests may potentially lie.

Despite their short existence, such laws have already aroused criticism. The main argument of the opponents to these laws is that the restrictions to the relationship between PCs and physicians may harm the advancement of research and of medicine in general [5].

The Israeli National Health Insurance Law [14] was amended in 2010 to require the annual disclosure to the Ministry of Health of payments or money’s worth exceeding 2500 NIS from manufacturers, importers or marketers of pharmaceuticals or medical equipment to medical personnel (physicians, nurses or pharmacists), and/or healthcare organizations, with both payers and their beneficiaries having to disclose the payment [15]. The list of payments is published annually on the Ministry of Health’s website [16]. Notably, the 2500 NIS-threshold for disclosing payments (approximately 685 US dollars in 2010, when the law was amended) is much higher than that required by transparency laws in other countries, such as France, in which any payment above 10 Euros requires disclosure, Portugal (25 Euros), and the U.S. (10 US Dollars). It was the legislator’s intent that the law would ensure maximum transparency and serve as an effective tool by the regulator for overseeing conflicts of interest in healthcare in order to ensure the most efficient allocation of public funding sources for the good of the patient population [17].

The aim of the present study was to examine if this law had achieved its goal some 4 years after it was passed. Our specific aims were to examine the impact of the law on the stakeholders in the Israeli health system and pharmaceutical industry, to identify the law’s shortcomings and difficulties in its implementation, and to propose further courses of action in this field.

## Methods

### Stakeholder interviews

Between July 2012 and July 2013 we conducted 42 in-depth interviews (refer to Table 1 for the main questions asked during the interview) with 46 representatives of relevant stakeholders: the Israeli Ministry of Health (10 representatives), HMOs (9), PCs (9), senior physicians and leaders of medical associations (11), patient organizations and health reporters (7). The interviewees were identified using the snowball sample method [18]. Each interview lasted an hour on average and dealt with the effects and extent of the legislation and industry self-regulation, as well as the interviewees’ attitudes and opinions about the best way to regulate the relationship between physicians and PCs.

**Table 1:** Main questions of the semi structured interview

1.	In your opinion, how should the relationship between physicians and PCs be managed, and to what extent does the legislation in this issue follow the right direction?
2.	In the last few years, where there changes in the conduct of PCs and their beneficiaries; what type of changes?
3.	A question to representatives of PCs: In the last few years what actions did you take regarding the relationship between the company and physicians? What does the company intend to do in the future?
4.	In the last two years, has the relationship between physicians and PCs decrease/expand/did not change? Please specify.
5.	In the last two to three years did the characteristics of medical research and conferences you have visited change?
6.	To what extend are the members of the health basket committee involved with technology manufacturers (funding of travel to conferences, lectures, counselors, etc.)?
7.	Are any writers of guidelines related in any way to manufacturers of technologies that are included in these guidelines? How many?
8.	To what extent are decision makers in HMOs (including pharmaceutical services and medical directors, purchase managers) involved with commercial companies or bodies?
9.	Are lecturers at professional conferences given guidance to disclose their relationship with pharmaceutical companies at the beginning of a lecture?
10.	In activities organized by patient organizations in the last year, how much of the activities were funded by the patient organization itself and who was involved in funding the difference between the actual cost of the activities and the sum of money given by the patient organization?
11.	Was there a change in the practice of giving physicians products with PCs’ logos? To what extent was the observed change? Is there employer supervision on this practice?
12.	Was there a change in the characteristics of visit of PCs’ representatives to HMO physicians? Is there supervision in this issue?
13.	Who should supervise the relationship between PCs, physicians and their organizations?
14.	How should the behavior in this relationship be regulated (legislation, self-regulation)?
15.	To what extent should the legislator be involved in the relationship between PCs and physicians?

The interviews were subjected to thematic analysis using the Narralizer software (http://www.narralizer.com). Through data analysis, we adopted a positioning perspective, which assumes that interviewees’ professional and organizational identities influence and in many ways determine their attitudes on this topic.

### Descriptive analysis of payment reports

For additional context, we reviewed reports to evaluate the law’s impact on disclosure of payments by PCs and their beneficiaries. To that end, we descriptively analyzed the Ministry of Health’s payment report for the years 2011 to 2012 [16], the records of the Israeli PC conglomerate - Pharma Israel (2011–2012) [19], and the Israeli Medical Association (IMA) report detailing payments received by IMA and other medical associations in Israel (2009–2012).

A recently published article on this issue [20] dealt with the legal perspective, including the roles of the law and the way in which legislation contributes to shaping policy and behaviour, as well as to the temporary provision that preceded the law. In addition, the roles of self-regulation were also presented. This article focuses on the relationship between doctors and pharmaceutical companies, and the ways to regulate it. In particular, we focus on the way these issues are perceived by the stakeholders.

Moreover, in this article we present an extensive analysis of the donation reports published by the Ministry of Health, Pharma Israel and the Israel Medical Association, with reference to the size of the donations and their objectives. This information makes it possible to examine the degree to which the legislation is implemented.

## Results

### Interviews with stakeholders

Several themes and subcategories were identified in the interviews. These are detailed below and summarized in Table 2.

**Table 2:** Examples of quotes supporting the major categories and subcategories

The need for a state law to regulate the relationship between PCs and physicians	
Subcategory	Examples of Quotes
Transparency	*“From the beginning the legislation was drafted around transparency in order to let the public judge. The goal was to change the norm, to get physicians to understand that receiving gifts has negative effects on them and to create a different culture based on disclosure. […] Publicity is the real deterrence.”*
The law has little to no influence on the relationship between PCs and physicians	
Subcategory	Examples of Quotes
Lack of awareness of the law	“*“… nobody has ever read that law”*”.
Loopholes in the law	*“The law is not transparent enough since you don’t need to disclose consulting for PCs. Everything can be called consulting. This is the place to hide things.”*
	*“Another way to remunerate a physician without disclosure is to fund a post-marketing study. These ‘studies’ are conducted after the drug is in the market and they interest no one. They are conducted only for marketing purposes. The company offers money to the physician in order to shift fifty patients to its drug and write a research report about it. Both the physician and the company benefit from the deal. The physician earns a lot of money for writing some brief report and gains experience with the drug, while the company shifts patients to its drug and also forges a relationship with the physician, which from now on becomes its ‘friend’.”*
An ambiguous definition of “payment”	*“There are many issues not addressed by the legislation. For example, if a patients’ organization organizes a conference and the company buys a stand, does this count as a payment or is it a business expense? And what about a situation when the company buys a stand or funds a conference like this but pays directly to the conference company in charge, instead of giving the money to the patients or physicians’ organization? The fact is that our accounting department will write it down as a business expense for all intents and purposes. […] The same applies if I send a physician to a conference but I pay the hotel or the conference fee myself, or if the headquarters abroad pays for it. In that case, the local branch of the company doesn’t spend a penny. […] We didn’t get answers to these type of questions and we also decided not to issue directives, so that each company acts the way it looks right to it and according to the directives it receives from its headquarters”.*
Lack of enforcement	*“As long as there is no enforcement, the law’s influence is reduced. I don’t see willingness to enforce the law at the Ministry of Health. In order to do that you need organizational prioritization of this issue, like assigning workers, budget. The law was not meant to deal with extreme cases, but with the everyday aspects of the relationship between physicians and PCs. If there is a complaint, the Ministry of Health with deal with it, but first you need someone to complain.”*
	*“The disclosure as it is today is a joke. The Ministry of Health doesn’t check if everybody that has to disclose indeed discloses. When we look at the report we discover a gap between the number of registered parties and the number of parties that reported. […] A further problem is that the report includes only sums of money and names. They don’t ask what the money was for. They don’t check if the sums are correct either.”*
Self-regulation versus state regulation	
Subcategory	Examples of Quotes
Self-regulation imposed by pharmaceutical company	*“I issued a directive in our company forbidding giving support to conferences spanning over weekends, unless physicians pay for the weekend from their own pocket. In the past, they would organize a conference from Thursday to Saturday: half a day of scientific content on Thursday, another two-hour meeting on Friday and then the whole weekend empty.”*
	*“Reps can give gifts under the following conditions: first, a cost limit of 10 dollars, and second, the gift must be related to the physician’s job. For example, we will not give a movie ticket, even though it costs less than 10 dollars.”*
	*“The multinational companies have very strict ethical rules. The local CEOs of these companies are salaried employees, so if a CEO breaches the company’s internal regulations, or even if there is a slight suspicion of infringement of these rules, he goes home. The CEOs of these companies are very minded to this issue and fear the headquarters. They would prefer to sell less and forgo revenues rather than take a risk and mess with the company’s ethical rules.”*
Self-regulation is stricter than state regulation	*“We have been publishing our payments in a European site for five years now, so for us the law didn’t have any influence. […] I can surpass the local regulation in any aspect.”*
Observed changes in the relationship between PCs and physicians	
Enactment of contractual relations between PCs and physicians	*“When I ask for support for the association from company X I have to sign a complex contract, which might get me and the association into trouble. This includes a commitment to return the money that we will not use for the conference. In the past, the association retained the change and this money served to fund the association’s activities. Different companies have different requirements. Our life is more difficult.”*
	*“A few years ago a rep came to my office and handled me an envelope containing a flying ticket to Rome for the launching of a new drug. This practice of giving flying tickets directly to physicians was common…. Today they [PCs] do not give tickets like that and if they want to fly a physician abroad, they have to turn to the hospital.”*

### Views on regulating the relationship between PCs and physicians

The interviewees held different and even opposing views on the appropriate way to regulate the relationship between physicians and PCs. While some favored self-regulation, others insisted on state regulation or some combination of both. Not surprisingly, these views tended to correspond with the interviewees’ organizational or professional affiliation (although there were some remarkable exceptions).

### Views on transparency of the relationship between PCs and physicians

Virtually all interviewees agreed that transparency is important to the relationship between PCs and physicians and they all approved of the goal of the law to enhance transparency. One of the promoters of the law explained:

*"From the beginning the legislation was drafted around transparency in order to let the public judge. The goal was to change the norm, to get physicians to understand that receiving gifts has negative effects on them and to create a different culture based on disclosure. […] Publicity is the real deterrence."*

None of the interviewees opposed the requirement of the law to disclose payments.

### Issues relating to implementation of the law

Most interviewees, regardless of professional or organizational affiliation, expressed doubts and reservations about the implementation of this law. In their view, it has very little to no influence on the payments and the norms of the relationship between PCs and physicians for the reasons described below.

#### Lack of awareness of the law

Of the 46 interviewees, as many as 18 were not aware of the existence of a law requiring the disclosure of payments by PCs, and none of the remaining interviewees claimed to be thoroughly familiar with it, although it was highly relevant to their professional activities. Some of the interviewees, who appeared to be aware of the law, showed little or no knowledge of its dispositions. Although this lack of awareness of the law and its details was more pronounced among physicians, it was also prevalent among senior managers and other stakeholders in the health system. An HMO manager scornfully remarked, *"… nobody has ever read that law"*. Although this statement seems to be an exaggeration, this attitude towards legislation about the relationship between physicians and PCs was expressed by most interviewees, albeit more subtly.

#### Loopholes in the law

Interviewees who were familiar with the law and its dispositions, agreed about its lack of effectiveness in restraining PCs’ payments and gifts to physicians. In their view, the law has many loopholes, thereby attracting “creative interpretations” aimed at bypassing it. For example, the law deals exclusively with payments, and ignores other forms of remuneration to physicians. Whilst all these forms of remuneration to physicians are legal and widely accepted in this field, as most of our interviewees attested, the main concern is that in some cases, they may be overpriced and used as a means to attain influence over a particular physician. For example, “consulting” is a form of remuneration that has evolved into a common practice among senior physicians and key opinion leaders. This practice can take many forms, and its goal is not restricted to exchange of knowledge, but also to promotion of PCs’ marketing [21]. The limits of “consulting” to PCs are loosely defined, thus making this practice an easy channel for payments to physicians without public scrutiny. As the CEO of a patients’ organization explained,

*"The law is not transparent enough since you don't need to disclose consulting for PCs. Everything can be called consulting. This is the place to hide things."*

Another form of remuneration to physicians that does not require disclosure under the law is payment for lectures. Many physicians, mostly key opinion leaders, are sought-after speakers by PCs organizing conferences and lectures. These key opinion leaders bestow an aura of respectability to PCs’ sponsored events, and therefore PCs are willing to pay large sums of money to have them lecture about their products. But as the CEO of a PC explained, these payments may serve other purposes as well:

*"PCs invite physicians to speak at conferences and pay them large sums of money in order to become 'friends'. For example, a company offers ten thousand shekels to a physician for one lecture. This same physician would have agreed to lecture for one thousand and five hundred shekels and the company knows it, but nevertheless the company offers him a bigger sum in order to become his 'friend'".*

Another way to “buy” senior physicians without disclosure to the Ministry of Health is to sponsor or even to create patients’ organizations and to appoint physicians to their boards. These physicians are paid for their service, but they receive the money from the patients’ organization and not directly from the PC, thus bypassing the law’s disclosure requirements. A still other form of remuneration is payment for funding of post-marketing or phase IV trials. Some interviewees attested that these studies have no real scientific interest and are mostly meant to shift patients to the drug made by the company funding the study. A PC’s CEO quoted above describes this method:

*"Another way to remunerate a physician without disclosure is to fund a post-marketing study. These 'studies' are conducted after the drug is in the market and they interest no one. They are conducted only for marketing purposes. The company offers money to the physician in order to shift fifty patients to its drug and write a research report about it. Both the physician and the company benefit from the deal. The physician earns a lot of money for writing some brief report and gains experience with the drug, while the company shifts patients to its drug and also forges a relationship with the physician, which from now on becomes its 'friend'."*

Finally, several interviewees mentioned that the law might be bypassed by business and accounting procedures, such as paying directly for the hotel and the conference fee when inviting a physician to attend conference abroad. Moreover, in the PCs’ view, these are business expenses, not payments, and thus do not require disclosure. This leads us to another shortcoming of the law, which is the lack of a clear-cut definition of a payment.

#### Ambiguous definition of payments

Although the law goes a long way to define the term “payment” as clearly as possible, some interviewees, mostly from PCs, complained that this definition is not clear enough and that it leaves some room for interpretation. A senior executive at a PC illustrated this problem in the case of PCs’ funding of conferences:

*"There are many issues not addressed by the legislation. For example, if a patients' organization organizes a conference and the company buys a stand, does this count as a payment or is it a business expense? And what about a situation when the company buys a stand or funds a conference like this but pays directly to the conference company in charge, instead of giving the money to the patients or physicians' organization? The fact is that our accounting department will write it down as a business expense for all intents and purposes. […] The same applies if I send a physician to a conference but I pay the hotel or the conference fee myself, or if the headquarters abroad pays for it. In that case, the local branch of the company doesn't spend a penny. […] We didn't get answers to these type of questions and we also decided not to issue directives, so that each company acts the way it looks right to it and according to the directives it receives from its headquarters".*

On the other hand, Ministry of Health officials dismissed this criticism and argued that the definition of payment is very clear. They also invited those who have questions to turn to the Ministry for clarifications and pointed to the fact that as to this moment there were no inquiries from any party requiring disclosure about this issue.

#### Lack of enforcement

Many interviewees cast doubts over the Ministry of Health’s willingness and capacity to enforce the law and pointed to the fact that there has been no enforcement yet. The main reason for this lack of enforcement was thought to be the Ministry’s weakness vis-à-vis other players in the health system. A senior official at one of the HMOs offered his point of view about this issue:

*"As long as there is no enforcement, the law's influence is reduced. I don't see willingness to enforce the law at the Ministry of Health. In order to do that you need organizational prioritization of this issue, like assigning workers, budget. The law was not meant to deal with extreme cases, but with the everyday aspects of the relationship between physicians and PCs. If there is a complaint, the Ministry of Health with deal with it, but first you need someone to complain."*

Lack of enforcement or even of minimal checking of fulfillment of disclosure obligations by all the relevant parties was noted, as a senior executive at an international PC remarked:

*"The disclosure as it is today is a joke. The Ministry of Health doesn't check if everybody that has to disclose indeed discloses. When we look at the report we discover a gap between the number of registered parties and the number of parties that disclosed. […] A further problem is that the report includes only sums of money and names. They do not ask what the money was for. They don't check if the sums are correct either."*

On the other hand, Ministry of Health officials explained that the lack of enforcement during the first years of the law is a deliberate policy whose goal is to learn from the data obtained and only then to start enforcing. Notably, the Ministry of Health has changed the disclosure framework and for the last several years so that the sum and general purpose of the payments have to be disclosed; however, many purposes are noted as “other” because they do not fall under any of the specified categories in the disclosure form.

#### Lack of deterrence

Interviewees from all sectors noted that the publication of payment sums does not deter the parties involved in the relationship from engaging in unethical conduct. In their view, transparency policies are not necessarily effective in instating norms of financial modesty and restrain the relationship between physicians and PCs. Moreover, transparency may achieve the opposite effect by leading to an “arms race” between physicians competing for industry money (in the same way that the publication of CEOs salaries fostered an “arms race” between them). As some interviewees said, “shame is long gone” in today’s health system and thus we cannot expect physicians to renounce payments from the pharmaceutical industry of their own accord.

### Attitudes to self-regulation

Most interviewees were aware that multinational PCs and their umbrella organizations have adopted strict ethical codes that regulate their relationships with physicians; that these codes were implemented both at the company and industry level and in most cases included enforcement mechanisms and sanctions to infringers. Interviewees were also aware that in addition to this self-regulation, medical associations and employers also enacted ethical codes and regulations regarding the relationship.

Our interviews indicated that despite the establishment of an ethical code between the PCs’ umbrella organizations and the IMA in this context, there were doubts concerning the code’s effectiveness in changing the norms of the relationship, due to insufficient implementation and enforcement. As the chairperson of a patients’ organization who is closely acquainted with this issue states,

*"the ethical code is a nice booklet, but it is not implemented in practice. […] There are no enforcement mechanisms. Everything depends on people's goodwill".*

### Observed changes in the relationship between PCs and physicians due to regulation

The self-regulatory measures brought a shift in the conduct of the relationship between PCs and physicians, which went from over-hospitality to contractual relations. This shift entailed restraining the gifts by bringing issues of transparency and accountability to the fore. For example, many PCs set restrictions to the distribution of gifts and samples to doctors. Moreover, the level of hospitality at medical conferences sponsored by PCs was reduced (including the common practice of paying for the expenses of the physician’s partner) and the non-academic contents (such as touristic activities and attractions) were mostly suppressed. A senior executive at a pharmaceutical company describes this change regarding conferences:

*"I issued a directive in our company forbidding giving support to conferences spanning over weekends, unless physicians pay for the weekend from their own pocket. In the past, they would organize a conference from Thursday to Saturday: half a day of scientific content on Thursday, another two-hour meeting on Friday and then the whole weekend empty."*

In the same vein, another executive explained their new policy on gifts to physicians:

*"Reps can give gifts under the following conditions: first, a cost limit of 10 dollars, and second, the gift must be related to the physician's job. For example, we will not give a movie ticket, even though it costs less than 10 dollars."*

The most significant change in the physician-PCs relationship appeared to be the enactment of contractual relations between PCs and physicians accepting favors. In contrast to past custom, in recent years all financial support from PCs to physicians and medical organizations is based on formal contracts. According to our interviewees, the contracts are strict and include minute detail of the activities being funded and the expenses for each activity. The chairperson of a medical society describes (and complains about) this shift in companies’ policy:

*"When I ask for support for the association from company X I have to sign a complex contract, which might get me and the association into trouble. This includes a commitment to return the money that we will not use for the conference. In the past, the association retained the change and this money served to fund the association's activities. Different companies have different requirements. Our life is more difficult."*

Another change is the funding of individual physicians’ travel to medical conferences through their employer or medical association and not directly like in the past. A senior hospital physician illustrates this change with a personal story:

*"A few years ago a rep came to my office and handled me an envelope containing a flying ticket to Rome for the launching of a new drug. This practice of giving flying tickets directly to physicians was common…. Today they [PCs] do not give tickets like that and if they want to fly a physician abroad, they have to turn to the hospital."*

The goal of this regulation was to cut off this channel of influence on physicians by taking the decision of who to send to the conference from the hands of PCs. However, many interviewees explained that PCs often find ways to circumvent this and choose which doctors will be benefitted. Still, they stated that the shift towards stricter standards in the relationship between physicians and PCs had a significant impact in Israel. At the company level, some interviewees indicated that company headquarters abroad are very strict towards their local branches in Israel on this issue, enforcing the company’s ethical standards and closely supervising their activities. Thus, the local branches of multinational PCs have strong incentives to comply with company standards, even if it reduces profitability. An HMO senior executive with close professional ties to the industry explains how this works:

*"The multinational companies have very strict ethical rules. The local CEOs of these companies are salaried employees, so if a CEO breaches the company's internal regulations, or even if there is a slight suspicion of infringement of these rules, he goes home. The CEOs of these companies are very minded to this issue and fear the headquarters. They would prefer to sell less and forgo revenues rather than take a risk and mess with the company's ethical rules."*

Additionally, because most patient organizations receive a large part of their budget from pharmaceutical companies they felt that the law affected their fundraising opportunities.

According to some interviewees, self-regulations enacted by HMOs and hospitals on the relationship between PCs and physicians, in addition to the industry self-regulations have brought significant changes in the conduct of the relationship in Israel that are in line with the international experience. These changes, be them cosmetic or profound, are the background on which the new legislation is supposed to act.

### Self-regulation is stricter than state regulation

Some interviewees remarked that the ethical codes and standards of multinational PCs are stricter than state regulation, since these internal codes need to comply with international standards. These standards, mainly from the European Union and the United States, precede Israel’s standards, making the legislation redundant, at least for these companies. A senior executive at a multinational PC provided an example of this redundancy regarding the central tenet of the Israeli legislation, transparency:

*"We have been publishing our payments in a European site for five years now, so for us the law didn't have any influence. […] I can surpass the local regulation in any aspect."*

### Participation of stakeholders in the legislation process

Some interviewees, mostly from the pharmaceutical industry and from patients’ organizations complained that they were excluded from the legislation process and that their voice was not heard, although they comprise the main sector affected by the law. This complaint was not just about matters of procedure or unfair treatment, but dealt with the essence of the legislation process itself. According to these interviewees, the law was promoted without a deep understanding of the needs and processes in the field. Moreover, some even argued that the law obeyed political motives which were characterized as populist. The CEO of a large multinational PC summed up this position:

*"The problem with the temporary order was that they didn't check its influence and they proceeded directly to legislation, without examining the situation in the field and without letting the relevant parties to express their views."*

### Analysis of payment reports

The Ministry of Health’s report included disclosure of payments by PCs totaling 29.3 million NIS in 2011 and 32.7 million in 2012, whereas recipients disclosed receiving 41.2 million NIS in 2011 and 41.4 million NIS in 2012. Over the 3-year period described in the report, there were discrepancies between the payments disclosed by donors and those disclosed by the beneficiaries. In addition, in many cases, the purpose of the payment was either not recorded or only vaguely described. For example, it is not clear if a purpose stated as “participation in a conference” constitutes payment for registration only or if it also includes the flight ticket and the hotel. Similarly, the purpose of “medical literature” may indicate paying for downloading a paper from a publisher’s website, which usually costs up to 50 dollars, or for medical books, which may cost hundreds of dollars.

Pharma Israel reported payments totaling 21.2 million NIS in 2011 and 20.4 million NIS in 2012. The major beneficiaries were hospitals (11.0 and 11.4 million NIS in 2011 2012 respectively) and patient associations (5.0 and 4.0 million NIS in 2011 and 2012, respectively). The most common purpose of payments was support of postgraduate training abroad (8.5 and 9.2 million NIS in 2011 and 2012, respectively).

The IMA includes 189 professional societies. Between 2009 and 2012, less than half of the societies (75/189, 40%) submitted at least one report detailing payments received from PCs, and only 16 societies (8.5%) submitted annual reports. Similar to the information obtained from the Ministry of Health’s report, there was marked variability in the sums of payments by PCs, with some societies receiving increasing sums of money between 2009 and 2012 and others receiving decreasing payments over the same period of time.

## Discussion

Three main findings emerge from the present study. First, virtually all interviewees agreed that transparency is important to the relationship between PCs and physicians and none of them opposed the requirement of the law to disclose payment.

Second, some 4 years after implementation of the law requiring the disclosure of payments from PCs to the health system, most interviewees reported to have witnessed a change in the regulatory climate of the PCs-physicians relationship; however this change was probably prompted mostly by industry self-regulatory measures rather than by state legislation. Although there were differences of opinion between interviewees regarding the scope and depth of these changes, most agreed that at least ostensibly the situation is different. The most significant change in the physician-PCs relationship appeared to be the enactment of contractual relations between PCs and physicians. In contrast to past custom, all financial support from PCs to physicians and medical organizations is currently based on formal contracts. According to our interviewees, the contracts are strict and include details of the activities being funded and the expenses for each activity. Contractual relations imply a different logic than gift exchanges. Exchange of gifts is a means to forge and sustain reciprocal social relationships, while concealing the economic interest in the exchange; gifts create reciprocal debt relationships that are seen as voluntary but are in fact obligatory [22]. In contrast, contractual relations are limited to the specific exchange at issue and do not foster any relationship or sense of obligation beyond the terms of the contract.

The third finding that emerges from the study is the pervasive feeling among the stakeholders that self-regulation is more effective than state regulation. One of the main advantages of self-regulation is that it draws its legitimacy from those involved in the relationship, instead of being imposed by the state. Some interviewees pointed out that the standards of international PCs are stricter than Israel’s standards, thus making the law redundant. Indeed, the law is not explicit and is open to interpretation. For example, if payments to medical personnel or to healthcare organizations are indirect. For example, if a PC pays the conference registration fees of several physicians by transferring the payment directly to the company organizing the conference, the law is not clear if the PC should disclose this payment.

The impact of the law on the behavior of individual physicians was claimed to be limited at best. Suggested causes were lack of awareness of the legislation, particularly among physicians; ambiguous definition of “payments” and loopholes in the legislation that attract other forms of remuneration to physicians and lack of enforcement of legislation. All these indicated that the disclosure requirement was not taken seriously enough, thereby harming the credibility and reliability of the Ministry of Health reports. Indeed, reports published by the Ministry of Health [16], Pharma Israel [19], and the IMA showed only some disclosure of payments by both donors and beneficiaries, as well as inconsistencies between the total payments disclosed by PCs and those disclosed by their beneficiaries. Although the Ministry of Health has changed the disclosure framework, the payment sum is disclosed according to general categories only (e.g. support of participation in Israeli conferences; support of travel and participation in conferences abroad; funding of medical equipment; partial funding of renovations; and “other”). Consequently, many payments fall under the category of “other” and are not specified in the report.

Self-regulation also has several shortcomings. First, there is always a risk of code infringement, especially in the absence of strong enforcement mechanisms including sanctions [23]. Our interviews confirmed the results of a previous study showing that there are doubts concerning the effectiveness of the IMA’s ethical code for changing the norms of the relationship between PCs and physicians, due to insufficient implementation and enforcement [24]. Second, self-sanctioned ethical codes may be used to bestow legitimation upon ethically controversial practices [25, 26]. Third, self-regulation is sometimes used to prevent state regulation [23].

Information on implementation of transparency laws relating to the relationship between PCs and physicians is lacking. A study in Vermont and Minnesota found that relevant data were hard to get; that loopholes in the law made it difficult to identify patterns of payment; that PCs often omitted physicians’ names from their disclosure reports; that there was no enforcement strategy; and that payments often exceeded the allowed limit of $100. The authors concluded that disclosure laws are not very effective to restrain PCs’ influence on physicians [27, 28]. Another study similarly found that PCs spend large sums on marketing to physicians; that the law did not eradicate the controversial practices from the relationship; and that it was not clear whether the disclosure of payments helped patients, since they usually do not look up the information about their doctors [29]. A study in Maine and West Virginia found that transparency laws had negligible to small effects on physicians’ prescribing behavior of statins and selective serotonin reuptake inhibitors [30]. In the USA, the ‘Open Payments’ database has been accessible to the public since 2014. Its aim is to report data about a wide range of financial interactions to present a comprehensive picture without passing judgment on what types of interactions lead to health care innovation and what may constitute potential conflicts of interest [31]. Many papers analyzing relations between pharmaceutical industry payments and physicians in various medical fields have been published over the past couple of years. It remains to be seen if and how such a database would affect the relations between PCs and physicians.

This study is not without limitations. The views represented in this study are specific to their 42 participants. The opinions voiced may have been those of the organization or company they represent rather than their own view on the issue at hand. However, we believe that the anonymity of the interviews allowed the participants to speak freely. We further believe that their positions as leaders in their fields make them appropriate representatives of their sectors and the type of reactions they may have to the legislation at hand.

## Conclusions and implications for policymaking

Despite the short period since the law was implemented in Israel, it has produced discernible changes in the relationship between PCs and practicing physicians and an increased awareness for the need of transparency; however, it is not clear if this change was caused by the law itself or if it is the outcome of self-regulatory measures of the pharmaceutical industry. We suggest that future efforts should focus on enforcement of the law and deterrence of violations, possibly by investigating discrepancies between the payment disclosed by donors and beneficiaries. Additionally, amendments should be made to the law in order to shut loopholes, and there should be periodic follow up of relevant databases and interviews of relevant stakeholders in order to seek feedback and identify problems in implementation.
